# Control of Bacterial Canker in Kiwifruit Using Botanical Products from *Adesmia balsamica* Bertero ex Colla in Kiwifruit cv. Hayward Orchards

**DOI:** 10.3390/plants14243726

**Published:** 2025-12-06

**Authors:** María Isabel Chávez, Martín Balladares, Jessica Ahumada, Jael Coloma, Paula Molina, Alejandro Madrid, Rolando Chamy, Katy Díaz

**Affiliations:** 1 Escuela de Ingeniería Bioquímica, Facultad de Ingeniería, Pontificia Universidad Católica de Valparaíso, Valparaíso 2340025, Chile; isabel.chavez@uv.cl (M.I.C.); martin.b.orellana@gmail.com (M.B.); jesahumadar@gmail.com (J.A.); 2 Farmacopea Chilena, Escuela de Química y Farmacia, Universidad de Valparaíso, Valparaíso 2360000, Chile; 3 Laboratorio de Pruebas Biológicas, Departamento de Química, Universidad Técnica Federico Santa María, Av. España 1680, Valparaíso 2390123, Chile; jaelceledonc@gmail.com (J.C.); pamolinae@gmail.com (P.M.); 4 Laboratorio de Síntesis Organica y Productos Naturales (LPNSO), Facultad de Ciencias Naturales y Exactas, Universidad de Playa Ancha, Leopoldo Carvallo 270, Playa Ancha, Valparaíso 2360000, Chile; alejandro.madrid@upla.cl

**Keywords:** resinous exudate, plants extracts, kiwi, *Pseudomonas syringae* pv. *actinidiae*

## Abstract

Global kiwifruit production has been severely affected by *Pseudomonas syringae* pv. *actinidiae* (*Psa*), which causes kiwifruit bacterial canker. The main strategies for controlling this pathogen involve the use of copper-based compounds and antibiotics, which are insufficient and promote the development of bacterial resistance. Therefore, this study evaluates the efficacy of different botanical products obtained from wild-grown and in vitro-grown plants, with 25–75% hydroalcoholic extracts (ethanol–water; 0.7 L/ha) effectively reducing the symptoms of the disease in kiwifruit, both in vitro and in vivo during a growing season. Additionally, field trials confirmed that the formulations promote better fruit yield and quality, demonstrating through acute oral toxicity testing in rats that the botanical product administered has no toxicity, making these botanical products a promising and sustainable alternative strategy for combating plant pathogen-induced diseases in an environmentally friendly manner.

## 1. Introduction

Kiwi fruit, a vitamin-rich fruit native to the Chinese Himalayas, is widely produced and grown around the world for its nutritional and economic value [1]. Global kiwi production has been on the rise, growing at an annual rate of approximately 3% over the last 10 years [2]. However, bacterial canker caused by *Pseudomonas syringae* pv. *actinidiae* (*Psa*) has been one of the most serious diseases affecting kiwis, mainly *Actinidia deliciosa* [A. chev] Liang et Fergurson and *A. chinensis* Planch., which has significantly reduced the yield and quality of kiwifruit in several countries around the world [3,4,5]. Chile, meanwhile, ranks third in the ranking with around 148,850 tons produced and 146,500 tons exported. However, it is estimated that the planted area has decreased, reaching 8576 hectares of production between green and yellow kiwis [6], due to adverse weather conditions (low temperatures) and the development of bacterial canker in kiwifruit. [7].

*Psa* can cause lesions on the bark, tissue necrosis, wilting, and, in severe cases, plant death [8]. During flowering, the inoculum can be spread by wind, rain, pollinators, and human activity, spreading from young plants to adult plants. Once an orchard is infected, rapid natural spread is expected [9], with symptoms generally developing on leaves in the spring and on buds and branches in fall, when weather conditions are most favorable for the development of the disease [10]. Given the high severity and damage caused by the disease, and the fact that there are periods when the plant shows no symptoms, preventive control is crucial due to the irreversibility of the damage caused by vascular necrosis in plants affected by *Psa* in kiwifruit [11]. Currently, there is no known cure for the disease, only some preventive control strategies. The most commonly used strategies to combat bacterial canker in kiwifruit include obtaining resistant varieties, proper orchard management, use of elicitors, and chemical or biological control methods. In this context, the application of plant defense inducers has gained relevance, as demonstrated by Nunes da Silva et al. [12], who showed that treatments with methyl jasmonate and salicylic acid activate key defense mechanisms in *A. chinensis* var. *deliciosa*, enhancing the plant’s ability to respond to *Psa*. Regulating humidity and avoiding overhead irrigation also helps reducing its spread. Other strategies include the preventive application of copper-based compounds and antibiotics [13,14], but they cause serious problems such as residues in fruit, damage to plants, toxicity to humans and other organisms, as well as inducing resistance mechanisms in *Psa* and other pathogens [13]. Therefore, it is essential to develop new alternative control strategies, such as biopesticides derived from natural products obtained from plants and microorganisms that offer significant potential in managing losses without compromising product quality [15].

Plants have the ability to synthesize a wide variety of secondary metabolites related to different defense mechanisms, such as alkaloids, terpenoids, and phenolic compounds, including flavonoids and tannins, many of which have antimicrobial properties [16] that are present in the secretory structures of many plant families, especially Fabaceae [17,18]. Among them are some species of the genus *Adesmia* (Fabaceae), endemic to the arid and semi-arid high Andean regions of Chile, Argentina, Bolivia, and Peru [19]. Some of the species studied belonging to the Balsamicae series are characterized by large amounts of resinous exudate in their branches and leaves [20]; they are known by the popular name of “Paramela” or “Jarilla” [21], among which the most studied species is *A. boronioides* Hook. f. whose essential oil is mainly used as an ingredient in infusions and in the cosmetics industry for its antioxidant properties and aromatic characteristics. Some types of sesquiterpene secondary metabolites, such as esquelenone and isoesquelenone, have been identified in the composition of the essential oil of the leaves [19,22,23]; On the other hand, the resinous exudate from the foliage also has antibacterial activity, whose bioactivity is justified by the presence of two bisnorsesquiterpenes (squel-6-en-9-one and squel-7-en-9-one) [24]. Another plant in the series, valuable for its biological properties, is *A. balsamica* Bertero Ex Colla. Chalcones, dihydrochalcones, and C-prenylated chalcones have been isolated and identified from the resinous exudate obtained from the foliage, notable for them in vitro anti-oomycete, anticancer, and antibacterial properties [25,26,27,28].

The demand for sustainable and environmentally friendly agricultural strategies is increasing, and naturally occurring pesticides can play an increasingly crucial role in this context, as they have the advantages of easy degradation in the environment, selective control, and safety for non-target organisms [29]. So far, our research has been based on experimental trials using plant products to evaluate in vitro antibacterial activity and validated under controlled greenhouse conditions, using molecular tools (qPCR; AriaMx Real-time PCR System, Agilent Technologies, Santa Clara, CA, USA) to determine their effectiveness in kiwi plants; however, there are no trials under field conditions in kiwi orchards. In this context, using botanical products based on resinous exudates and extracts has the advantage of better yield, rapid production, lower environmental impact, limited use of solvents, and reuse of these solvents for new production processes [28].

Therefore, the objective of this study was to evaluate the effectiveness and toxicity of different botanical products (extracts and resinous exudates) from wild-grown plants and in vitro-grown from specimens of *A. balsamica* on bacterial canker of kiwifruit and their effect on the yield and quality of fruit harvested in a kiwifruit orchard.

## 2. Results and Discussion

### 2.1. Extraction Yields and Phytochemical Characterization of Bioactive Compounds in Exudates and Extracts from Wild Plants and In Vitro of Adesmia balsamica

Table 1 shows the results of the extraction yields of secondary metabolites from the exudates and extracts of *A. balsamica*, both in wild plants and in vitro. The highest yield was obtained from the hydroalcoholic extract from in vitro plants (HIV) with 78% on a dry basis. In the case of wild plants, the highest yield was obtained from the ethanolic exudate (EEAB) (22%), followed by the hydroalcoholic extract (HAB) (14%), and finally the aqueous exudate (70AB) (3%). This is mainly due to the polarity of the solvent used and the fact that the compounds present in the plant are more nonpolar, which allows for higher extraction using ethanol as a solvent rather than water. In previous studies [28], work was done to obtain a resinous exudate from *A. balsamica* using dichloromethane as a solvent, which showed antibacterial activity against *Psa* at a concentration of 100 µg/mL. In this study, ethanol was used as the solvent, and the main secondary metabolites present in the ethanolic resinous exudate were identified. Four compounds were identified: compound **1**: 1-(2,4-dihydroxy-5-(3-methylbut-2-en-1-yl)phenyl)-3-phenylpropan-1-one (3.1%); compound **2**: Glabranine (2.9%); compound **3**: Isocordoin (3.9%); and compound **4**: 2′,4′-dihydroxychalcone (<0.1%) (Figure S1 and Table S1). They were identified by comparing their ^1^H and ^13^C NMR spectra with the reported work [28] and quantified by HPLC-PDA (Figure S2 and Table S2). These four compounds were found in the resinous exudate obtained with dichloromethane, but the proportion is different compared to the determination made in this study; this is due to the change in the polarity of the solvent used in the extraction of bioactive compounds. The main compound obtained with ethanol is compound **3**, with a yield of 3.9%, which is five times higher than that found in the dichloromethane exudate. In contrast, compound **4** had a yield of over 10% in the dichloromethane exudate, and a very low yield of less than 0.1% in the ethanol exudate. The four compounds are also found in hydroalcoholic extracts from wild plants and in vitro but in a lower percentage (Figure S2 and Table S3). It should be noted that the extraction yields of the major compounds found in the ethanolic resinous exudate may vary depending on the season and location where the *Adesmia* plant is extracted [30,31].

Table 1 shows the measurement of the total amount of polyphenols and flavonoids in wild plant and in vitro botanical products of *A. balsamica*, using ethanol and a mixture of ethanol and water (50:50 in wild plants and 25:75 in in vitro plants) as solvents. The highest percentage, both in terms of total polyphenols and flavonoids, was found in the resinous exudate, and this is because this botanical product is a concentrate of bioactive compounds extracted from the external part of the plant (resin). The hydroalcoholic extracts from wild plants and in vitro plants show similarity in the measurements of total polyphenols, at 6%, but the amount of flavonoids is higher in wild plants (13.9%). The aqueous exudate was not measured for total polyphenols and flavonoids due to low extraction yield.

Studies on phytochemicals with antimicrobial and antioxidant properties show great promise for suppressing plant diseases and, therefore, reducing the application of synthetic chemical pesticides. There are several reports in which bioactive compounds have been detected and identified in plant species. For example, in *Guizotia abyssinica* L. leaves, the polyphenol content (total phenols and flavonoids) was evaluated in methanolic and aqueous extracts. The results show that the amount of total polyphenols is 6% for methanolic extracts and 10% for aqueous extracts, while the amount of total flavonoids is 60% for methanolic extracts and 20% for aqueous extracts. The presence of these secondary metabolites (flavonoids) would be associated with the antioxidant and antimicrobial capacity of these extracts [32]. Similarly, analysis of hydroalcoholic extracts from the leaves and fruits of *Smilax aspera* L. revealed the presence of phytochemicals, which exhibit strong antibacterial activity against *Erwinia amylovora*, *P. syringae* pv. *actinidiae*, and *Xanthomonas campestris* pv. *campestris*, a property attributed to their rich composition of flavonoids and other secondary metabolites, which exhibit anti-inflammatory and cytotoxic properties [33]. Numerous studies have reported consistent results, indicating that polyphenols derived from pomegranate, as well as various plant extracts, significantly reduce swimming motility in different phytopathogenic bacteria. This behavior is attributed to the action of phenolic compounds present in high concentrations, which are capable of interfering with interbacterial communication (quorum sensing) and altering the integrity of the extracellular matrix, promoting its denaturation and thereby affecting key processes associated with colonization and virulence [34].

Analysis of the ethanolic resinous exudate revealed the presence of four compounds that are prenylated derivatives of flavonoids, mainly of the chalcone class. All these compounds exhibit varied biological activity, particularly antibacterial activity [28,35,36].

### 2.2. In Vitro Antibacterial Activity

Kiwifruit Bacterial Canker is a serious disease that limits industrial kiwi production worldwide. Therefore, this study evaluated the efficacy of different botanical products such as resinous exudates and extracts produced from the foliage of wild *A. balsamica* collected from central Chile, specifically from Valparaíso, Curauma; and other extracts produced from plants grown in vitro, against *Psa* through in vitro and in vivo bioactivity tests on kiwi orchards.

Of the four botanical products evaluated using the serial microdilution test, high inhibition of *Psa* growth was determined when testing the plant extract in vitro with an MIC value of 100 µg/mL. However, the extract obtained from wild *A. balsamica* plants showed the same sensitivity as the ethanolic resinous exudate with a minimum inhibitory concentration (MIC) value of 200 µg/mL, being less sensitive than the extract prepared in water at 70 °C with a value of 800 µg/mL (see Table 1). This lower inhibitory capacity can be attributed to the fact that the secondary metabolites present in solution are more polar because the extraction process is carried out only with hot water at a temperature of 70 °C for a short immersion time, which does not allow the extraction of all the other more apolar compounds such as prenylated chalcones, which are known to have antibacterial properties.

The minimum inhibitory concentrations (MIC) of various ethnobotanical extracts have been classified by Holetz et al. [37] as “good” antibacterial activity below 100 µg/mL; from 100 to 500 µg/mL, moderate; from 500 to 1000 µg/mL, weak; and above 1000 µg/mL, inactive. Therefore, the in vitro plant-based botanical product has “good,” outstanding activity according to this classification, and two of our botanical products can be classified as having moderate MIC, which is a remarkable result in the control of phytopathogenic microorganisms.

Plants in the Balsamicae series, to which *A. balsamica* belongs, are rich in bioactive compounds, such as flavonoid derivatives, which are responsible for their antibacterial activity. The mechanism of action is attributed to the alteration of the bacterial membrane by prenylated chalcones, as demonstrated in our previous study [28]. In that work, we confirmed that the isolated compounds (specifically **1** and **4**) possess significant individual antibacterial activity against *Psa* through membrane disruption. However, in the present study, while these major compounds were quantified, the efficacy observed in the extracts likely results from a synergistic interaction among these major chalcones and other minor constituents (‘entourage effect’), rather than the action of a single isolated molecule [38]. This multi-target strategy is a well-documented phenomenon in complex plant extracts applied in postharvest treatments, where minor compounds can enhance the bioavailability or stability of the active principles [39]. Furthermore, the ability of phenolic acids to act at the cytoplasmic level on Gram-negative bacteria has also been demonstrated. The partially lipophilic nature of phenolic acids allows them to cross the cell membrane by passive diffusion, lowering the pH value in the cytoplasm. By lowering the pH value and altering the structure of the cell membrane, they cause protein denaturation, thus compromising the integrity of the membranes [40].

Currently, the control methods used against *Psa* are the adoption of Good Agricultural Practices and the use of bactericidal compounds based on copper compounds and antibiotics. However, the emergence of *Psa* strains resistant to copper and antibiotics, together with evidence of phytotoxicity caused by these compounds [41,42], has led to the search for new alternatives for controlling this pathogen. Additionally, we can conclude that the in vitro effect of the positive control (copper sulphate) generates moderate inhibition, with 640 µg/mL greater than the results of HIV, HAB and EEAB, which is why these botanical products are more effective against the pathogen in vitro. To date, there are several studies on the experimental use of different botanical products such as essential oils, plant extracts, and antibacterial resinous exudates against different pathogens [43,44], which have several advantages over synthetic pesticides as they are environmentally friendly, less expensive to produce, have low toxicity to wildlife and humans, and are biodegradable in the field when applied to the soil.

Various research teams have studied different effective and environmentally sustainable strategies to combat *Psa* infections, such as the application of elicitors to enhance the natural resistance of plants [45,46], the creation of new biological agents [47], and the use of natural compounds with antibacterial properties [48,49,50,51,52].

Consequently, our in vitro results warrant field validation to confirm the efficacy of these botanical products against *Psa*. Such studies would demonstrate, for the first time, their potential to prevent damage caused by this phytopathogen in kiwifruit orchards.

### 2.3. Effectiveness of Psa Control Under Field Conditions

Each treatment proved effective in reducing *Psa* load and decreased damage to fruit yield in field trials, also showing promising results in terms of its effectiveness in preventing bacterial canker in kiwi plants.

The application of preventive organic compounds could reduce the establishment of a pathogen, preventing the development of disease and damage to the environment. Copper compounds applied at the right concentration and at the right time can help prevent *Psa* infection, but this remains a challenge, as their use affects the natural environment. Regarding the incidence and severity of damage to Hayward kiwifruit leaves in both evaluations, all treatments showed moderate incidence, with leaves displaying symptoms of the disease (Table 2), the botanical product based on hydroalcoholic extracts (25% ethanol-75% water) from plants grown in vitro applied at a dose of 0.7 L/ha (T3) and 1.4 L/ha (T4) managed to reduce the incidence and severity of bacteriosis damage by 10% in leaves of kiwi cv. Hayward in field conditions. The effects of these extracts (T3 and T4) exhibiting a significantly lower incidence than the negative control treatment T0, just like positive control T8 (with copper sulfate pentahydrate). Likewise, these treatments showed the lowest level of severity, also differing statistically from the negative control treatment.

This effect may be explained by the capacity of the phenolic constituents in the hydroalcoholic extracts to activate specific defense-related processes in plant tissues and to directly impair pathogen viability. Beyond their broad antimicrobial reputation, several phenolic subclasses, such as ellagitannins, flavonols, and phenolic acids are known to disrupt key physiological functions in bacterial pathogens [53,54]. These compounds can destabilize the integrity of the cytoplasmic membrane by interacting with membrane-bound proteins and phospholipids, ultimately generating pore formation, ion leakage, and depolarization of the proton motive force [28,50]. In *Xanthomonas* and other Gram-negative phytopathogens, phenolics interfere with quorum-sensing signaling molecules and suppress the expression of virulence-associated genes, particularly those governing motility, extracellular polysaccharide production, and type III secretion system activity [49,52]. Such targeted disruptions extend beyond simple growth inhibition by attenuating the pathogen’s ability to colonize host tissues and overcome plant defenses, thus providing a mechanistic basis for the strong antibacterial activity observed in this study. In this regard, essential oils from cinnamon bark and oregano encapsulated in organic polymers have shown to be an interesting alternative for protecting kiwi plants from *Psa* [55]. However, their low solubility, chemical instability, and extraction cost limit their inclusion in commercial formulations. Therefore, the plant extracts tested in this study are a viable alternative since they do not present disadvantages in this regard, are soluble and stable, and have a low extraction cost.

Regarding the incidence and severity of damage to buds and flowers of kiwi cv. Hayward, experimental applications were evaluated, where samples of EEAB applied at 0.7 L/ha (T1), HIV at 1.4 L/ha (T4), HAB at 0.7 L/ha (T5) and 1.4 L/ha (T6), and 70AB at 1.4 L/ha (T7) showed a significant reduction in ha (T1), HIV at 1.4 L/ha (T4), HAB at 0.7 L/ha (T5) and 1.4 L/ha (T6), and 70AB at 1.4 L/ha (T7) reduced the damage caused by bacteriosis in flower buds (Table 3). According to literature, the use of contact bactericides, such as copper products, is recommended in the early stages of flower bud development to control *Psa* in the field [56]. However, our results show that the extracts have a lower incidence on flower buds compared to the use of copper sulfate pentahydrate at 0.6 g/L (T8), so again the ethanol-water extract shows a lower incidence value. This enhanced efficacy may be related to the fact that the formulations were derived from plants produced under in vitro conditions, where environmental variables are tightly regulated. Such controlled growth environments not only prevent exposure to pathogens and abiotic stressors [57] but also modulate metabolic pathways in ways that differ substantially from field-grown plants. In particular, in vitro culture is known to influence the biosynthesis and accumulation of phenolic compounds by altering nutrient availability, hormonal balance, and light conditions—factors that directly regulate secondary metabolism. Numerous studies have shown that tissue-cultured plants often accumulate higher and more stable levels of phenolic compounds compared with conventionally grown material [58]. As a result, plants cultivated in vitro frequently exhibit a more consistent and enriched profile of specific bioactive metabolites, including phenols with well-established antimicrobial and antioxidant activities [59]. This metabolite enrichment could therefore contribute to the superior biological performance observed in the formulated products [60]. This makes hydroalcoholic extracts more effective antimicrobials than those obtained from wild plants, which could be applied preventively to obtain better results at the time of flowering.

### 2.4. Evaluation of Parameters Based on Kiwifruit Orchard Yield and Quality

The data were recorded in a single harvest 5 months after the last application. Table 4 shows the values of the different parameters evaluated in each of the treatments. The plants were treated with different formulations, obtaining statistically significant differences in kiwi quality and productivity yield. However, the plants sprayed with HAB at 1.4 g/L had the highest fresh weight fruit, reaching an average of 111.1 g of the total harvested, while plants sprayed with copper sulfate pentahydrate 0.6 g/L showed the lowest fruit weight values, with an average of 98 g of the total harvested (Figure S3a). In the Hayward variety, fruits are larger than those of other varieties [61,62], so the ideal size would be that of the fruits in treatment T6. It is worth mentioning that Shan et al. [63], in their study on the effect of pesticides on kiwi quality, concluded that fruit size did not increase significantly with an increase in the concentration of chemical pesticides, thus recommending their use within a reasonable range. In contrast, in our study, a higher concentration of botanical products (potential biopesticides) was used, obtaining better results in terms of weight per fruit.

The effect of each treatment on fruit volume was determined by measuring the ellipsoid, and there was no significant variation between the different treatments. However, the fruits from T6, which received 1.4 g/L HAB, stood out, showing a significant increase in fruit volume compared to the other treatments (Table 4).

In evaluating the number of fruits per tree, a significant difference was observed between treatments, with treatment T4 (hydroalcoholic extract at 1.4 g/L) standing out over T1 (ethanolic exudate at 0.7 g/L). These findings suggest that a higher concentration of hydroalcoholic extract increases fruit production, probably due to the greater availability of bioactive compounds in higher doses, which stimulates fruiting and protects flowering. On average, treatment T4 produced 236 fruits per tree, surpassing T6 (195 fruits) and, to a lesser extent, T8 (152 fruits). The use of plant extracts has been shown to be effective in enhancing plant growth and yield by positively influencing physiological processes, promoting both cellular activity and the production of plant hormones [64]. The increase in flavonoids and polyphenols present in the exogenously applied extracts could have increased the number of cells by increasing cell division and elongation. Seaweed extract has also been reported to increase grape fruit length [65] and, at a concentration of 3000 ppm, to advance ripening, total yield, and improve the post-harvest quality of kiwifruit [66].

The lowest °Brix values (10–11) correspond to hydroalcoholic extracts at different concentrations (T3 and T4) from in vitro plants. According to the minimum maturity parameters for the start of the kiwi harvest established by the kiwi committee, we should consider whether it is seasonal fruit from 5.0° to 6.0° Brix and whether it is early fruit of 4.8° to 5.5°Brix [67]. In both cases, these are considered low values, so T3 and T4 decrease the sugar concentration in the fruit, improving ripening times. According to marketing terms, dry weight would be a more accurate tool than soluble solids concentration alone for controlling minimum harvest quality and complying with export and import requirements [68]. In this case, hydroalcoholic extracts from wild plants (T5 and T6) achieved the highest dry weight and fresh weight compared to the positive control treatment (copper sulfate) (Table 4). This indicates that these formulations could be more efficient in ensuring better yield and quality, which is crucial for meeting the demands of the domestic and international markets.

Yield was calculated in tons per hectare of harvested kiwifruit, assuming a planting density of 800 trees/ha (Table 4). Preventive control of *Psa* with treatments based on *A. balsamica* extracts produced a higher annual fruit yield compared to the positive control treatment (copper sulfate), demonstrating that they do not alter pre-harvest physiological processes. On the contrary, they maximize kiwi production, sustaining the profitability of the fruit grower’s business. The yield was also calculated at the national level, assuming an area of 7.900 ha. The results reported by Patiyal et al. [69] show that a very high fruit load can have negative effects on the quality of kiwifruit. However, based on our data, treatment T4 suggests that the use of this formulation would be effective against *Psa* and would enhance its effect on the harvest.

These results demonstrate the potential of this plant species as a source of bioactive molecules with antibacterial properties, in particular demonstrating its value as a new botanical product for the development of future biopesticides. It is therefore essential to evaluate acute oral toxicity in order to clarify the safety profile of the bioactive extracts tested.

### 2.5. Acute Oral Toxicity of the Active Extract in Rats

In relation to the body weight of the animals recorded weekly during the study, it was determined that the first group showed an average weight increase of 14.1%, while the second group had an average increase of 10.5% (Table S4). No mortality was recorded in either group of rats administered the 2000 mg/kg b.wt. dose (Table 5). During the observation period, no clinical signs associated with the administration of the product were detected.

In relation to macroscopic pathologies in the first group, mild alterations were observed at necropsy, such as mild pulmonary and hepatic congestion. In the second group, the findings were limited to mild hepatic congestion (Table S5). Histopathological analysis of the tissues showed mild periportal hepatic sinusoidal congestion in the liver. In the lung, moderate multifocal subacute to chronic interstitial pneumonia was observed. Finally, moderate diffuse interstitial congestion was observed in the kidney (Table S6). Finally, the results indicate that the median lethal dose (LD_50_) is >2000 mg/kg for the toxicity test, with a cutoff dose = 5000 mg/kg. These results classify the product as Category V according to the Globally Harmonized System (GHS), which means that it cannot be harmful if swallowed. Category III according to the World Health Organization (WHO), which is low hazard, and Category 5 according to the Organization for Economic Cooperation and Development (OECD).

In the case of other botanical products, such as clove oil and eugenol, they have been classified as minimal risk pesticides by the United States Environmental Protection Agency (EPA-US) and products containing them are exempt from the requirements of the Federal Insecticide, Fungicide, and Rodenticide Act (FIFRA) [70]. Various acute and chronic toxicity studies of clove oil have reported an oral LD_50_ of 3597.5 mg/kg b.wt., and no adverse effects have been observed in subchronic toxicity tests, with a non-observed adverse effect level (NAOEL) of 900–2000 mg/kg/day. The oral LD_50_ of eugenol has been reported to be between 2650 and 3000 mg/kg b.wt, which is consistent with the results of our active plant extract against *Psa* [71,72]. Similarly, in a study of extracts from calendula inflorescence residues, flavonoids administered at a dose of 5000 mg/kg b.wt. for 14 days did not produce any abnormal clinical symptoms or mortality in standard rats and mice from the Institute for Cancer Research Mouse (ICR; both sexes, *n* = 5) [73]. Unlike, the positive control used in in vivo tests, which has an oral LD_50_ of 300–2000 mg/kg, according to manufacturer’s data (AGROCUPPER^®^, ANASAC, Santiago, Chile).

This is the first reported study of the acute oral toxicity of a bioactive ethanolic extract from *A. balsamica*, whose composition is mainly secondary metabolites from the phenol and flavonoid groups. The results allow this botanical product to be classified as harmless and sustainable, contributing to the growing field of natural product chemistry and offering an alternative to synthetic chemicals in the agricultural industry.

## 3. Materials and Methods

### 3.1. Plant Material

This study used the aerial parts (leaves and stems) of the *A. balsamica* Bertero ex Colla plant from Curauma, Valparaíso Province, V Region, Chile (latitude 33°08′49.9″ S and longitude 71°34′20.8″ W), extracted between July 2023 and January 2024. A voucher specimen (VALPL1899) was deposited at the VALP Herbarium, department of Biology, Universidad de Playa Ancha, Valparaiso, Chile.

#### Description and Growing Conditions of the Orchard

The evaluation of the botanical products was carried out during the season from September 2023 to April 2024 in a commercial orchard of kiwi cv. Hayward (*A. deliciosa* cv. *Hayward*), positive for *Psa*, located in the town of Teno, province of Curicó, Maule Region, Chile, with a southern latitude of 35°2′27.18″ and a longitude of 71°11′0.70″, planted in 1993 with a planting distance of 4.0 × 2.5 m, conducted in a Spanish Parron system. The experiment was carried out in four rows of the orchard, with 108 plants per row. Each treatment (T0–T8) was sprayed in a targeted manner (11 seg./plant) with a backpack sprayer on 12 plants to prevent drifting of the formulation. In each row, groups of three plants were randomly divided. The doses were calculated by extrapolating the number of liters applied per plant according to the planting pattern of the orchard, using a spray volume of 1000 L/ha. Climate data’s were obtained from a weather station near the site where the study was conducted. The minimum and maximum temperatures and precipitation during the period in which the trial was conducted can be seen in Table S7, and the record of temperatures and precipitation at the time of application of the treatments can be seen in Table S8.

### 3.2. Preparation of Exudates and Extracts from Wild-Grown and In Vitro-Grown Plants of Adesmia balsamica

The preparation of exudates and extracts from *A. balsamica* was carried out according to the procedure described by [28], with modifications. For the ethanolic exudate of *A. balsamica*, 1 kg of fresh plant material and 9 L of ethanol (Merck. S.A, Santiago, Chile, 99% purity) were used. The solution obtained was filtered and concentrated in a rotary evaporator R-100, (BUCHI Corporation, New Castle, DE, USA), at a temperature of 50 °C until a resinous exudate was obtained. From this resin, a stock solution with a concentration of 50 g/L (EEAB) was prepared, which was diluted in water to prepare ethanolic exudate solutions with concentrations of 0.7 g/L and 1.4 g/L. For the aqueous exudate, 1 kg of fresh plant material and 6 L of water at 70 °C for 45 s were used. The solution was then cooled and filtered. The concentration obtained was 2 g/L (stock solution (70AB)), which was diluted in water to obtain a concentration of 1.4 g/L.

To prepare the hydroalcoholic extract, 1 kg of chopped fresh plant material (3–5 cm) and 7 L of ethanol–water solution (50:50) were used for the wild plant and ethanol–water (25:75) for the in vitro plant. It was left to stand for 3 days, covered, in darkness, at room temperature. The solution obtained was filtered and then concentrated in a rotary evaporator (BUCHI, R-100, Flawil, Switzerland) at a temperature of 50 °C until a hydroalcoholic extract solution with a concentration of 20 g/L was obtained (stock solution, wild plant (HAB), and in vitro plant (HIV)). From this solution, solutions with concentrations of 0.7 g/L and 1.4 g/L were prepared by diluting with water. Table 6 shows the concentrations of the different formulations applied in the field, based on wild and in vitro *A. balsamica* plants.

The seeds of *A. balsamica* were collected in the Curauma sector, Placilla de Peñuelas, Valparaíso Region (Chile). The material was disinfected in a 1.0% NaOCl solution (prepared from Clorox^®^ 5.25% sodium hypochlorite) for 20 min, followed by three rinses with sterile distilled water. The seeds were sown in MS medium [74] supplemented with 3% (*w*/*v*) sucrose and solidified with 2.7 g/L Phytagel^®^; the pH was adjusted to 5.7 before sterilization. The cultures were incubated under a photoperiod of 16 h light/8 h darkness at 22 ± 2 °C. The seedlings obtained in vitro were used as explants (nodal segments) and subcultured in the same medium in order to increase the plant biomass for the preparation of hydroalcoholic extracts.

### 3.3. Isolation and Separation of Major Compounds in Botanical Products from Adesmia balsamica

To isolate the bioactive compounds from the ethanolic resinous exudate, preparative liquid chromatography was performed using a system equipped with a dual-detector array (PLC 2250, Gilson, Middleton, WI, USA), following the procedure described by [75] with modifications.

Thirty grams of resinous exudate were used to prepare a suspension with silica gel (40–36 µm mesh, Merck S. A, Santiago, Chile) and dichloromethane (99% purity, Merck S.A, Santiago, Chile) as the solvent. Once the solvent was evaporated, the resulting powder was loaded into a 50 mL syringe and placed atop a 330 g SiliaSep™ cartridge (SiliCycle, Quebéc, QC, Canada). Elution was performed using a gradient of hexane:ethyl acetate 99% purity (Merck S.A, Santiago, Chile,) from 100:0 to 0:100, at a flow rate of 145 mL/min. The wavelengths monitored were 254, 280, 323, and 365 nm. A total of 355 fractions (20 mL each) were collected. Each fraction was monitored by TLC (Silica gel 60 F_254_, Merck S. A, Santiago, Chile) using hexane:ethyl acetate (20:80 *v*/*v*) as the mobile phase. Fractions exhibiting similar profiles were combined and concentrated under reduced pressure using a rotary evaporator R-100(BUCHI Corporation, New Castle, DE, USA). Compound 1 was obtained from fractions 210–240; compounds **2** and **3** were obtained from fractions 244–272; and compound **4** was obtained from fractions 290–320.

To obtain pure compounds, a second separation step was carried out for each of the collected fractions using a SiliaSep™ 40 g cartridge (SiliCycle, Quebéc, QC, Canada) with a hexane:ethyl acetate gradient (100:0 to 0:100) at a flow rate of 20–40 mL/min. The compounds were identified using a Nuclear Magnetic Resonance Spectrometer (Avance 400 Digital NMR, Karlsruhe, Germany; 400 MHz for ^1^H-NMR and 100 MHz for ^13^C-NMR) with deuterated chloroform 99.8% purity (Sigma-Aldrich, Santiago, Chile) as the solvent.

The quantification of the major compounds in the botanical products was performed using the method described in [76], with modifications. A Shimadzu Prominence LC-20A series HPLC-PDA system (Kyoto, Japan) was used, consisting of a DGU-20A in-line solvent delivery and degassing unit, a CTO-20AC column oven, a SIL-20AC automatic sampler, an SPD-M20A diode array detector, and a system controller running LabSolutions software, version 4.42. Separation was achieved on a Luna C18 column (250 mm × 4.6 mm, 5 µm; Phenomenex, Torrance, CA, USA) at 30 °C. The mobile phase consisted of solvent A: acetonitrile (Supelco, ≥99% purity) and solvent B: water with 1% formic acid (Merck, 99% purity), used in gradient mode. The elution program was as follows: 0–12 min, 30–32% A; 12–16 min, 32% A; 16–25 min, 35% A; 25–35 min, 50% A; 35–40 min, 55% A; 40–50 min, 60% A; 50–75 min, 70% A; 75–80 min, 50% A; 80–85 min, 40% A; 85–90 min, 30% A; followed by a 5 min re-equilibration period, for a total run time of 95 min. The flow rate was 1 mL/min, the injection volume was 20 µL, and the detection wavelength was 220 nm.

The purity of compounds **1**–**4** obtained from the isolation of the ethanolic resinous exudate was determined by HPLC-PDA (Table S2 and Figure S2a). These isolated compounds were subsequently used as standards to construct calibration curves for the quantification of these metabolites in plant exudates and extracts (Table S2).

### 3.4. Measurement of Total Polyphenols and Total Flavonoids in Botanical Products from Adesmia balsamica

To measure the total polyphenol content in botanical products, the Folin–Ciocalteu colorimetric method was used, according to the modified procedure described by [75]. To 500 µL of sample, 2 mL of 10% Folin–Ciocalteu reagent (Merck, USA) was added. After 6 min, 2.5 mL of 7.5% sodium carbonate solution (Sigma-Aldrich, Santiago, Chile, 99.5% purity) was added, stirred, and kept in the dark for 1 h. Absorbance was measured at a wavelength of 765 nm using a spectrophotometer (Synergy H1, Biotek, Winooski, VT, USA). Gallic acid (40–600 mg/L) (Sigma-Aldrich, Santiago, Chile; ≥99% purity) was used as the standard. The measurements were performed in triplicate, and the result was expressed as g gallic acid in 100 g of sample. To determine the amount of total flavonoids, the colorimetric method based on aluminum chloride [75] was used, with modifications. To 600 µL of sample, 1.5 mL of 0.85% *w*/*v* sodium nitrite solution (Merck, Santiago, Chile; ≥98% purity) was added. After 5 min, 200 µL of 10% aluminum chloride solution (Merck, Santiago, Chile; 99% purity) was added; then after 8 min, 2 mL of a basic solution of 0.65 M sodium hydroxide (Sigma-Aldrich, Santiago, Chile; 99% purity) and ethanol (1:4) (Merck, Santiago, Chile; 99% purity) were added. Absorbance was measured at a wavelength of 510 nm using a spectrophotometer (Synergy H1, Biotek, Winooski, VT, USA). Quercetin (20–400 mg/L) (Sigma-Aldrich, Santiago, Chile; ≥99% purity) was used as the standard. The measurements were performed in triplicate, and the result was expressed as g quercetin in 100 g of sample.

### 3.5. In Vitro Bioactivity Test

#### 3.5.1. Bacterial Strain

The pathogenic strain of *Psa* used was *Psa*-190817, isolated from diseased plants of *A. chilensis* var. *deliciosa* (cv. Hayward) (Talca, Maule Province, Chile) and deposited in the culture collection of the Chilean Agricultural and Livestock Service [77]. This strain had shown greater resistance in previous antibacterial activity bioassays [28], which is why it was selected for this study. The bacterial strain was grown in a nutrient medium supplemented with 3% sucrose (SNA) and incubated at 27 °C for 24 h. The bacterial inoculum was produced in DIFCO^®^ Mueller-Hinton (MH) culture medium in glass test tubes and incubated at 27 °C in an orbital shaker at 150 rpm in the dark for 24 h and adjusted to a concentration of 1 × 10^8^ colony-forming units (CFU) × mL^−1^.

#### 3.5.2. *In Vitro Antibacterial Activity*

The antibacterial activity of the resinous exudates and extracts was determined by the microdilution method according to [28]. A stock solution was prepared at a concentration of 2000 µg/mL, from which a series of dilutions (range 1–1600 µg/mL) was made in a 96-well microplate with MH culture medium. Four controls were used: negative control 1 (only MH liquid culture medium inoculated with bacteria); negative control 2 (only substance plus solvent, 1% ethanol) without inoculating bacteria; and a blank control (only MH liquid culture medium, without inoculation). Copper sulfate at the same concentrations tested was used as a positive control; each well of the microplate was inoculated with 1.5 µL; it was then incubated at 35 °C for 24 h. Each treatment was tested in triplicate, and each reported value represents the mean ± SD of two independent experiments. The percentage of bacterial inhibition (PBI) was calculated based on the absorbance (DO) readings at 600 nm obtained using a Thermo Scientific Multiskan GO spectrophotometer (Waltham, MA, USA). One hundred µL of each concentration were transferred to a Petri dish to observe the presence or absence of pathogen growth (*Psa*) after 24 h. To obtain the MIC, the lowest concentration of compound at which no visible growth was observed in the Petri dishes was considered.

### 3.6. Evaluation of the Effectiveness of Botanical Products Against Disease

The treatments were applied during budding, from October to November, approximately every 10 days, completing a total of six applications. All applications were made on the same dates for each treatment, using a 15 L STIHL^®^ SR 450 motor sprayer. Nine different treatments were used: a negative control (T0; only water); EEAB 0.7 g/L (T1); EEAB 1.4 g/L (T2); HIV 0.7 g/L (T3); HIV 1.4 g/L (T4); HAB 0.7 g/L (T5); HAB 1.4 g/L (T6); 70AB 1.4 g/L (T7); copper sulfate pentahydrate 0.6 g/L (T8).

Two evaluations were carried out, one at the sprouting stage and the other at the flowering stage. The incidence and severity of the disease on the leaves was evaluated, marking 12 random shoots per experimental unit: expressed as a proportion, using the following Formulas (1) and (2):

(1) Incidence:(Number of diseased leaves/Total number of leaves evaluated)

**Figure 1:**
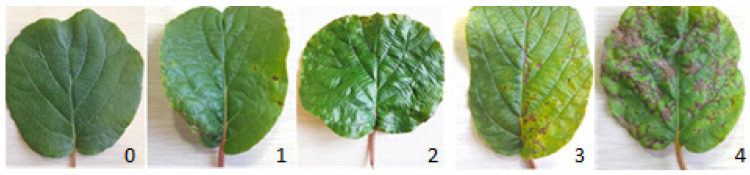
Descriptive scale according to severity for kiwi leaves. 0 = no spots (healthy), class 1 = 1 to 3 spots/leaf (incipient), class 2 = 4 to 10 spots/leaf (mild), class 3 = 11 to 25 spots/leaf (moderate), class 4 = 26 or more spots/leaf (severe).

Four classes or categories of leaf spot severity were defined according to [78], as follows: class 0 = no spots (healthy), class 1 = 1 to 3 spots/leaf (incipient), class 2 = 4 to 10 spots/leaf (mild), class 3 = 11 to 25 spots/leaf (moderate), class 4 = 26 or more spots/leaf (severe), which express the index of damage to the leaves (Figure 1).

And expressed as a proportion, using the following formula:

(2) Damage Severity Index:(n×v)/(V×N)

where **n** is the number of leaves per degree, **v** is the degree of damage, **N** is the total number of leaves observed, and **V** is the maximum range of the scale.

For the evaluation of the flowering stage, only the incidence of the disease was assessed as the number of affected flowers out of the total number evaluated. To do this, 200 flowers were randomly selected from the central tree of each repetition. In addition, the number of damaged buds was quantified in relation to the total number of buds evaluated per treatment. To do this, 100 flower buds were randomly selected from the central tree of each treatment, considering 25 of these for each quadrant of the plant, to quantify the number of those with necrosis and obtain the percentage of damaged buds per plant.

### 3.7. Evaluation of Kiwi Quality and Yield

Quality and yield were evaluated in the harvest carried out in April 2024. For this purpose, the central plant of each treatment per row was selected, from which six fruits were randomly selected and collected per experimental unit. Once a total of 24 kiwis per treatment had been collected, they were placed in separate labeled bags to be transported to the laboratory for refrigeration and storage for 24 h for evaluation and determination of the following parameters. For the quality evaluation, the fresh weight of each kiwi was determined by weighing each fruit individually on a precision scale (PX224 Ohaus Corporation; Parsippan, NJ, USA). To determine the dry weight of each fruit, a 2–3 mm thick slice was cut from the equatorial part (including skin and seeds) and placed on a tray to be dried in an incubator (MEMMERT; Schwabach, Germany) at 60 °C for 48 h. To evaluate commercial size, the distribution scale based on the weight range for the Hayward cultivar, produced by FRUSAN S.A. export company was used [79]. The geometric volume of each kiwifruit was calculated using Formula (3) for the volume of an ellipsoid:

(3) $$V=\frac{4}{3}\times \pi \times a\times b\times c$$

where, using a digital caliper, the major equatorial diameter, minor equatorial diameter, and polar diameter were measured in mm to obtain and consider the radius of the three measurements (**a**), (**b**), and (**c**) in cm.

To quantify the concentration of soluble solids (CSS), the Brix degrees (°Brix) of each fruit were determined by extracting 2–3 drops of kiwi juice using a hand press and dropping them onto the prism of the digital refractometer for measurement (HI96801, Hanna Instruments, Limena, Italy). To determine the yield per ton of kiwi production in Chile, the average number of fruits obtained per plant during harvest and the average fresh weight of kiwis per treatment were considered, as a result of applying the different formulations evaluated in the orchard. A planting density of 800 trees per hectare was assumed, with an approximate area of 7.900 hectares nationwide.

### 3.8. Statistical Analysis

The experimental design was arranged in completely randomized blocks (CRB) with nine treatments and four replicates. The data obtained were analyzed using In-foStat version 2020 (Córdoba, Argentina). To compare the treatments, the means of the leaf incidence assessment were subjected to a general linear and mixed model analysis (MLMix). The means of the severity evaluations on leaves and buds were subjected to a generalized linear mixed model analysis (MLG-Mix). When significant differences were detected between treatments (*p*-value < 0.05), data adjustment was performed using Fisher’s LSD multiple comparison test with a 95% confidence interval.

### 3.9. Acute Oral Toxicity of the Active Extract

The study was conducted in accordance with the OECD (Organization for Economic Cooperation and Development) Principles of Good Laboratory Practice ENV/MC/CHEM (98)17, in accordance with the Study Plan, based on the procedures described in Guideline 423 [80]. Healthy young female Sprague-Dawley rats approximately 8 to 12 weeks of age with a starting weight not exceeding ±20% of the mean weight (226 g) were used, and they were reared using the outbreeding system. They were housed in a cage in accordance with current NIH (National Institute of Health, Bethesda, MD, USA) standards for the proper maintenance of laboratory animals, at 22 ± 3 °C, with relative humidity between 30 and 70%, a light–dark photoperiod of 12/12 h, respectively, and were provided with food and water ad libitum. All animals were fasted for approximately 12 h before administration of the active ethanolic extract (HAB 1.4 g/L) and were provided with water only. In all experimental studies, each treatment consisted of three animals that received a single dose of 2000 mg/kg b.wt. in two consecutive stages, except for the control group, which was only given water, following the guidelines of OECD Guideline 423 for estimating the LD_50_. The experiment lasted 28 days.

The ethanolic extract that controlled bacteriosis in the kiwi orchard trial was used. Due to its physicochemical characteristics, the substance was administered directly, without the use of a vehicle. The dose was calculated proportionally to the weight of each animal and administered orally using a rigid orogastric tube with a balloon tip to ensure complete administration of the dose (Table 7). The dosing procedure was carried out identically in the first and second stages.

The animals’ survival was assessed twice a day throughout the study period to ensure continuous monitoring of their vital status. After administration of the compound, clinical toxicological evaluations were performed at 30 min, 4 h, and daily during the 14 days of observation. Each animal was examined outside its cage to assess its general condition, skin and coat, eyes, nasal cavity, oral cavity, abdomen, and external genitalia. Respiratory rate was also recorded, and abdominal palpation was performed to detect possible abnormalities, such as the presence of pathological masses (Table S9). In addition, food and water consumption was monitored daily through visual inspections, recording any changes in normal intake patterns. Complete necropsies were performed on all euthanized animals, including an external macroscopic examination of all natural orifices, organs, or tissues of the cranial, thoracic, abdominal, pelvic, neck, and skeletal cavities to record all observed abnormalities. In addition, tissue samples from selected organs of the animals that survived the first 24 h were sent to the laboratory. These samples were processed for histological sections and microscopic analysis, with the aim of obtaining additional information relevant to the study.

## 4. Conclusions

In this study, we evaluated the possibility of using botanical products obtained from wild and in vitro-grown *A. balsamica* plants to protect kiwifruit against *Psa*. Botanical products obtained from exudates and extracts are antibacterial under in vitro and field conditions. Products derived from hydroalcoholic extraction (ethanol–water) from plants grown in vitro and also from wild plants were effective in inhibiting the growth of *Psa*, thereby increasing fruit yield and quality. This can be attributed to their phytochemical composition, which contains a considerable percentage of polyphenols and flavonoids. Finally, the oral toxicity test in rats did not record any mortality when administered, making these plant extract-based formulations a very promising strategy for protecting kiwifruit, with a reduction in the disease index throughout the season. Although further research is needed to optimize the application conditions and treatment formulation (timing, number of applications, dosage) to improve the protection of kiwifruit and/or other crops, this work is one of the few that attempts to provide information under real operational conditions.

## 5. Patents

Patent Application Number 202501468, filed on 16 May 2025, with the National Institute of Industrial Property (INAPI), Chile. Title: Botanical product based on *A. balsamica* that prevents and controls diseases in kiwi plants. PCT/CL2025/050053.

## Figures and Tables

**Table 1:** Evaluation of extraction yield, total polyphenol content, total flavonoid content, and antibacterial activity in different botanical products based on wild *Adesmia balsamica* plants and in vitro.

Botanical Products	% Extraction Yield, on a Dry Basis	% Total Polyphenols ^a^	% Total Flavonoids ^b^	MIC (µg/mL)	Bacterial Inhibition (%)
EEAB	22	25 ± 0.25	38 ± 0.30	200	90
HIV	78	6.1 ± 0.10	5.0 ± 0.08	100	95
HAB	14	6.3 ± 0.08	13.9 ± 0.10	200	40
70AB	3	--	--	800	80
C (+)	-	-	-	640	28

a: Total polyphenols expressed as g of Gallic Acid in 100 g of sample. b: Total flavonoids expressed as g of Quercetin in 100 g of sample. (--): Not measured due to low extraction yield %. (-): Not applicable.

**Table 2:** Incidence and severity of damage to Hayward kiwi leaves two months after sprouting.

Treatment	Incidence *	Severity of the Disease *
T0	0.89 ^ab^	0.49 ^bc^
T1	0.88 ^ab^	0.48 ^bc^
T2	0.89 ^ab^	0.56 ^a^
T3	0.81 ^c^	0.43 ^cd^
T4	0.86 ^b^	0.41 ^d^
T5	0.90 ^a^	0.50 ^d^
T6	0.89 ^ab^	0.49 ^bc^
T7	0.87 ^ab^	0.52 ^ab^
T8	0.71 ^d^	0.24 ^e^
*p*-valor	<0.0001	<0.0001

* Different letters indicate significant differences (*p*-value > 0.05). PCM LSD Fisher.

**Table 3:** Incidence of damage to buds and flowers of kiwi cv. Hayward two months after bud break.

Treatment	Incidence of Late Blight on Flower Buds (*)
T0	0.35 ^a^
T1	0.24 ^b^
T2	0.40 ^a^
T3	0.30 ^a^
T4	0.22 ^b^
T5	0.23 ^ab^
T6	0.18 ^b^
T7	0.23 ^b^
T8	0.26 ^b^
*p*-valor	<0.0001

(*): Different letters indicate significant differences (*p*-value > 0.05). PCM LSD Fisher.

**Table 4:** Evaluation of quality and production performance parameters following the application of different botanical products for the control of *Pseudomonas syringae* pv. *actinidiae* on a kiwi orchard (January 2024).

Treatment	Number of Fruits (Unit)	Fruit Volume (cm^3^)	Fruit Fresh Weight (g)	°BRIX	Fruit Slice Dry Weight (3 mm) (g)	Tons/ha
T0	192 ± 49.10 ^ab^	89.9 ± 14.42 ^b^	99 ± 8.03 ^bc^	12.2 ± 0.40 ^a^	1.42 ± 0.22 ^c^	120,320 ± 32,938 ^abc^
T1	113 ± 73.72 ^b^	78.9 ± 12.08 ^c^	103.1 ± 13.03 ^abc^	11.6 ± 0.92 ^bc^	1.47 ± 0.15 ^c^	74,128 ± 49,496 ^c^
T2	137 ± 41.01 ^b^	94.3 ± 1.8 ^ab^	107.3 ± 1.69 ^abc^	11.7 ± 0.73 ^abc^	1.58 ± 0.20 ^bc^	93,087 ± 28,693 ^bc^
T3	174 ± 91.01 ^ab^	92.7 ± 9.03 ^b^	99.0 ± 14.27 ^bc^	11.2 ± 0.43 ^cd^	1.54 ± 0.26 ^bc^	114,306 ± 73,976 ^abc^
T4	236 ± 82.46 ^a^	94.9 ± 13.85 ^ab^	107.6 ± 13.10 ^ab^	10.9 ± 0.60 ^d^	1.63 ± 0.10 ^bc^	156,656 ± 42,567 ^a^
T5	132 ± 73.35 ^b^	96 ± 8.5 ^ab^	109.5 ± 10.22 ^ab^	12.1 ± 0.59 ^ab^	1.89 ± 0.12 ^a^	94,457 ± 55,919 ^bc^
T6	195 ± 90.56 ^ab^	103 ± 26.15 ^a^	111.1 ± 18.66 ^a^	12.1 ± 0.78 ^ab^	1.88 ± 0.15 ^ab^	132,913 ± 50,476 ^ab^
T7	147 ± 78.02 ^b^	86.9 ± 16.96 ^bc^	98.5 ± 18.64 ^bc^	12.4 ± 0.91 ^a^	1.75 ± 0.10 ^ab^	86,5,97 ± 41,634 ^abc^
T8	152 ± 34.71 ^ab^	86.4 ± 2.36 ^bc^	98.3 ± 2.34 ^c^	11.3 ± 1.10 ^cd^	1.60 ± 0.15 ^bc^	94,777 ± 23,000 ^bc^

Different letters indicate significant differences (*p* < 0.05).

**Table 5:** Mortality of animals dosed with 2000 mg/kg of the active extract of *Adesmia balsamica* after 28 days of oral administration.

Dose Level (mg/kg)	Females (Dead/Total)	Time of Death
2000 (1)	0/3	-
2000 (2)	0/3	-
Control	0/3	-

Control: animals without administration of the active extract. (-): No time of death (days, hours).

**Table 6:** Concentration of different formulations based on wild *Adesmia balsamica* plants and in vitro, applied in the Kiwi orchard located in the town of Teno, Curicó, Chile.

Treatment	Type of Plant	Type of Formulation	Concentration, in g/L
T0	-	Negative Control (only water)	0
T1	Wild	EEAB: Ethanolic Exudate	0.7
T2	Wild	EEAB: Ethanolic Exudate	1.4
T3	In vitro	HIV: Hydroalcoholic Extract (25:75 = ethanol–water)	0.7
T4	In vitro	HIV: Hydroalcoholic Extract (25:75 = ethanol–water)	1.4
T5	Wild	HAB: Hydroalcoholic Extract (50:50 = ethanol–water)	0.7
T6	Wild	HAB: Hydroalcoholic Extract (50:50 = ethanol–water)	1.4
T7	Wild	70AB: Aqueous exudate	1.4
T8	-	Positive Control (Copper sulfate pentahydrate)	0.6

**Table 7:** Animals dosed with 2000 mg/kg b.wt. (group 1) and 2000 mg/kg b.wt. (group 2) with concentrated plant extract (HAB 1.4 g/L).

N° Animal	Sex	Weight on Day 0 (g)	mg of Compound	mL of Compound
1 (group 1)	Female	204	408	0.41
2 (group 1)	Female	248	496	0.50
3 (group 1)	Female	200	400	0.41
1 (group 2)	Female	229	458	0.47
2 (group 2)	Female	223	446	0.45
3 (group 2)	Female	251	502	0.51

Body weight was recorded on day 0 before dosing, day 7 after dosing, day 14 after dosing, day 14 after dosing, and immediately before euthanasia to assess weight variation during the trial.

## Data Availability

The original contributions presented in the study are included in the article/Supplementary Material, further inquiries can be directed to the corresponding authors.

## Supplementary Materials

The following are available online at https://www.mdpi.com/article/10.3390/plants14243726/s1. Figure S1. Structure of secondary metabolites extracted from the ethanolic resinous exudate of *A. balsamica*. Figure S2: HPLC-DAD chromatogram (a) Standards of the major compounds of the *A. balsamica* plant, at a concentration of C1 = 465 mg/L (time ret. = 60.8 min); C2 = 277 mg/L (time ret. = 57.1 min); C3 = 255 mg/L (time ret. = 63.5 min); and C4 = 255 mg/L (time ret. = 9.6 min); (b) Ethanolic resinous exudate from wild plant; (c) Ethanol–Water = 50:50 extract from wild plant, and (d) Ethanol–Water = 25:75 extract from in vitro plant. Figure S3. Histopathological analysis of tissues from animals dosed with the product (concentrated plant extract) at 2000 mg/kg after 28 days. (a) H&E lung. Mild focal thickening of alveolar walls; (b) H&E lung. Focal area of tissue consolidation; (c) H&E liver. No histological alterations; (d) H&E kidney. No histopathological alterations. The blue arrow indicates the observation. H&E: methodology applied with hematoxylin eosin. Image captured at 100×. Scale 100 µm. Table S1: Structural elucidation of natural compounds. Table S2. Equation of the straight line and correlation factor of calibration curves for total polyphenols and flavonoids, by UV-VIS spectrophotometry, and for the major compounds C1 to C4, by HPLC-PDA. Table S3. Mass percentage of the major compounds C1 to C4, by HPLC-PDA, in the different botanical products. Table S4: Initial body weight of animals dosed with 2000 mg/kg of active concentrated extract. Group 1 and group 2. Table S5: Evaluation of body weight of animals treated with 2000 mg/kg of active extract 7 and 14 days after administration. Table S6: Monthly arithmetic mean of minimum temperature, maximum temperature, thermal oscillation, and monthly accumulated precipitation during the trial period. Table S7: Record of temperatures and precipitation on the day of application; Table S8: Evaluation of macroscopic pathologies (necropsy) in animals treated with 2000 mg/kg of active extract after 28 days. Table S9: Summary of clinical signs evaluated in rats.
