# Supplementary material for: Control of Bacterial Canker in Kiwifruit Using Botanical Products from Adesmia balsamica Bertero ex Colla in Kiwifruit cv. Hayward Orchards
Source: Plants (Basel). 2025 Dec 6;14(24):3726. doi: 10.3390/plants14243726 (PMC12736877; doi:10.3390/plants14243726)
Supplement: Supplementary file 1 [file plants-14-03726-s001.zip › plants-3986317-supplementary.pdf]

plants

MDPI

Supplementary Materials: **Figure S1**. Structure of secondary metabolites isolated from the ethanolic resinous exudate of *A. balsamica*. **Figure S2:** HPLC-DAD chromatogram a) Standards of the major compounds of the *A. balsamica* plant, at a concentration of C1=465 mg/L (time ret.=60.8 min); C2=277 mg/L (time ret.=57.1 min); C3=255 mg/L (time ret.=63.5 min); and C4=255 mg/L (time ret.=9.6 min); b) Ethanolic resinous exudate from wild plant; c) Ethanol:Water=50:50 extract from wild plant, and d) Ethanol:Water=25:75 extract from *in vitro* plant. **Figure S3.** Histopathological analysis of tissues from animals dosed with the product (concentrated plant extract) at 2000 mg/kg after 28 days. a) H&E lung. Mild focal thickening of alveolar walls; b) H&E lung. Focal area of tissue consolidation; c) H&E liver. No histological alterations; d) H&E kidney. No histopathological alterations. The blue arrow indicates the observation. H&E: methodology applied with hematoxylin eosin. Image captured at 100x. Scale 100µm. **Table S1**: Structural elucidation of natural compounds. **Table S2.** Equation of the straight line and correlation factor of calibration curves for total polyphenols and flavonoids, by UV-VIS spectrophotometry, and for the major compounds C1 to C4, by HPLC-PDA. **Table S3.** Mass percentage of the major compounds C1 to C4, by HPLC-PDA, in the different botanical products. **Table S4**: Initial body weight of animals dosed with 2000 mg/kg of active concentrated extract. Group 1 and group 2. **Table S5**: Evaluation of body weight of animals treated with 2000 mg/kg of active extract 7 and 14 days after administration; **Table S6**: Monthly arithmetic mean of minimum temperature, maximum temperature, temperature range, and monthly accumulated precipitation during the trial period; **Table S7**: Record of temperatures and precipitation on the day of application; **Table S8**: Evaluation of macroscopic pathologies (necropsy) in animals treated with 2000 mg/kg of active extract after 28 days. **Table S9**: Summary of clinical signs evaluated in rats.

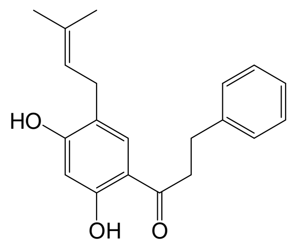

1-(2,4-dihydroxy-5-(3-methylbut-2-en-1-yl)phenyl)-3-phenylpropan-1-one (1)

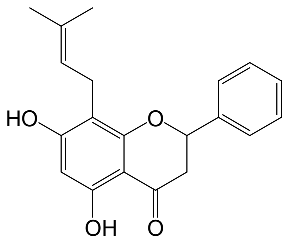

Glabranine (2)

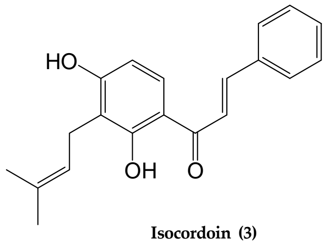

Isocordoin (3)

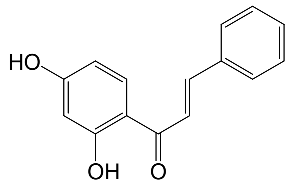

2',4'-dihydroxichalcone (4)

Figure S1. Structure of secondary metabolites isolated from the ethanolic resinous exudate of *A. balsamica*.

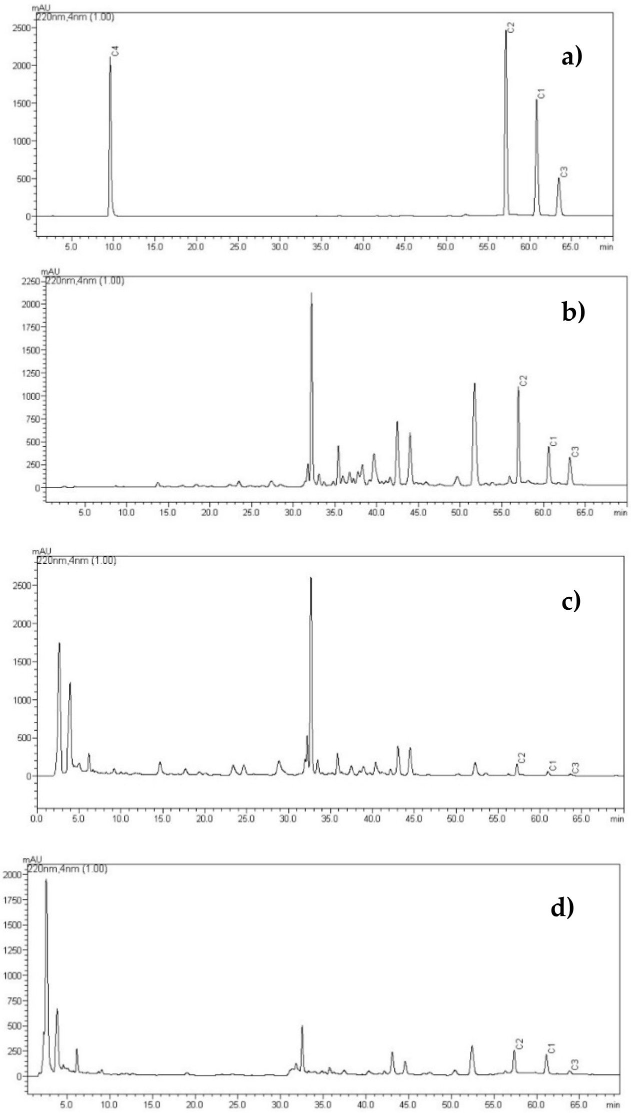

Figure S2: HPLC-DAD chromatogram a) Standards of the major compounds of the *A. balsamica* plant, at a concentration of C1=465 mg/L (time ret.=60.8 min); C2=277 mg/L (time ret.=57.1 min); C3=255 mg/L (time ret.=63.5 min); and C4=255 mg/L (time ret.=9.6 min); b) Ethanolic resinous exudate from wild plant; c) Ethanol:Water=50:50 extract from wild plant, and d) Ethanol:Water=25:75 extract from *in vitro* plant.

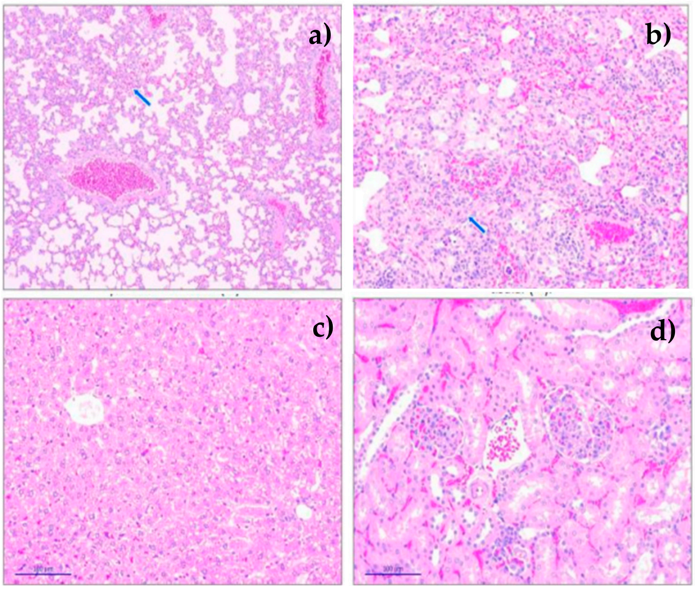

Figure S3. Histopathological analysis of tissues from animals dosed with the product (concentrated plant extract) at 2000 mg/kg after 28 days. **a)** Lung H&E. Mild focal thickening of alveolar walls; **b)** Lung H&E. Focal area of tissue consolidation; **c)** Liver H&E. No histological alterations; **d)** Kidney H&E. No histopathological alterations. The blue arrow indicates the observation. H&E: methodology applied with hematoxylin eosin. Image captured at 100x. Scale 100µm.

Table S1: Structural elucidation of natural compounds

Compound	^{1}H NMR (400 MHz, $CDCl_3$), δ/ppm:	^{13}C NMR (100 MHz, $CDCl_3$), δ/ppm:
**1: 1-(2,4-dihydroxy-5-(3-methylbut-2-en-1-yl)phenyl)-3-phenylpropan-1-one**	12.58 (1H, s, OH); 7.43 (1H, s,H-6′); 7.43-7.20 (5H, m, B ring protons); 6.43 (1H, s,H-3′); 5.94 (1H, s,OH); 5.26 (1H, m,H-2´´´); 3.26 (1H, m,H-2); 3.22 (1H, s,H-1′′′); 3.03 (1H, m,H-3); 1.77 (1H, s,H-4′′′); 1.77 (1H, s,H-5′′′).	203.5; 163.7 161.4; 141.0; 135.4; 131.4; 128.6; 128.6; 128.4; 128.4; 126.4; 121.4; 118.8; 113.6; 103.9; 39.8; 30.5; 29.1; 25.8; 17.9
**2: Glabranine**	11.98 (1H, s, OH); 7.42 (5H, m, B ring protons); 6.18 (1H, s, OH); 6.03 (1H, s,H-6); 5.42 (1H, dd,H-2, J= 3.0 and 12.9 Hz); 5.22 (1H, t, H-2′′, J= 7.2 Hz); 3.33 (2H, d,H-1′′, J= 7.2 Hz); 3.05 (1H, dd, H-3, J= 12.9 and 15.1 Hz); 2.85 (1H, dd, H-3. J= 3.0 and 17.1 Hz); 1.73 (3H, s, H-4′′); 1.63 (3H, s, H-5′′).	196.2; 163.7; 162.2; 159.6; 138.7; 135.1; 128.8; 128.8; 128.7; 128.7; 125.9; 121.5; 106.1; 103.2; 96.9; 78.9; 43.3; 25.8; 21.8; 17.8
**3: Isocordoin**	13.79 (1H, s, OH); 7.89 (1H, d, J=15.4 Hz, H-2); 7.74 (1H, d, J=8.9 Hz, H-6′); 7.60 (1H, d, J=15.4 Hz, H-3); 7.43 (5H, m, B ring protons); 6.43 (1H, d, J=8.8 Hz, H-5′); 6.15 (1H, s, OH); 5.31 (1H, t, J=7.1 Hz, H-2′′′); 3.48 (2H, d, J=7.1 Hz, H-1′′′); 1.85 (3H, s, H-4′′′); 1.78 (3H, s, H-5′′′)	192.1; 163.9; 161.7; 144.2; 136.0; 134.8; 130.6; 129.3; 129.3; 128.9; 128.9; 121.0; 120.5; 114.5; 114.1; 114.0; 107.9; 25.83; 21.75; 17.95
**4: 2′,4′-dihydroxychalcone**	13.38 (1H, s, OH); 7.91 (1H, d, J=15.9 Hz, H-2); 7.87 (1H, s, H-3′);7.84 (1H, d, J=8.4 Hz, H-6′); 7.57 (1H, d, J=15.9 Hz, H-3); 7.43 (5H, m, B ring protons); 6.43 (1H, d, J=8.4 Hz, H-5′); 5.92 (1H, s, OH).	191.9; 166.4; 162.7; 144.6; 134.7; 132.0; 130.7; 128.9; 128.5; 120.2; 107.8; 103.8

Table S2. Equation of the straight line and correlation factor of calibration curves for total polyphenols and flavonoids, by UV-VIS spectrophotometry, and for the major compounds C1 to C4, by HPLC-PDA.

**Analysis**	**Standard**	**Concentration range, in mg/L**	**Equation of the line, y = mx + b**	**Correlation factor, r^2**
Total Polyphenols	Gallic Acid, Merck, purity ≥ 99 %	60-480	y = 0.0033x + 0.0927	0.9966
Total Flavonoids	Quercetin, Sigma-Aldrich, purity ≥ 99 %	40-400	y = 0.0006x+ 0.0425	0.9999
Compound 1	Purity 98.0%**	58-465	y = 24119x - 158772	0.9998
Compound 2	Purity 98.5%**	34-277	y = 147373x + 693890	0.9985
Compound 3	Purity 98.5%**	32-255	y = 126193x + 384719	0.9982
Compound 4	Purity 98.0%**	32-255	y = 119436x + 451089	0.9990

(**), The compound obtained in the separation was purified, and its purity was determined by HPLC-PDA.

Table S3. Mass percentage of the major compounds C1 to C4, by HPLC-PDA, in the different botanical products.

Botanical products	%C1	%C2	%C3	%C4
Ethanolic Exudate / Wild Plants	3.1	2.9	3.9	< 0.1
Hydroalcoholic Extract Ethanol:water = 25:75 / *In vitro* Plants	0.58	0.21	0.18	< 0.1
Hydroalcoholic Extract Ethanol:water = 50:50 / Wild Plants	0.34	0.1	0.1	< 0.1
Aqueous Exudate / Wild Plants	--	--	--	--

%C: g of compound in 100 g of sample

(--): was not evaluated due to low extraction performance.

Table S4: Initial body weight of animals dosed with 2000 mg/kg of active concentrated extract. Group 1 and group 2.

N° animal	Sex	Weight Day 0	mg of compound	mL of compound
**Group 1**				
**1**	Female	204	408	0.41
**2**	Female	248	496	0.50
**3**	Female	200	400	0.41
**Group 2**				
**1**	Female	229	458	0.47
**2**	Female	223	446	0.45
**3**	Female	251	502	0.51

Group 1 and Group 2 correspond to the administration of step 1 and step 2, respectively.

Table S5: Body weight assessment of animals treated with 2000 mg/kg of active extract 7 and 14 days after administration.

N° animal	Sex	Weight on day 0 (g)	Weight on day 7 (g)	Difference in week 1 (g)	Weight on day 14 (g)	Difference in week 2 (g)
**Group 1**						
1	Female	204	224	20	233	9
2	Female	248	260	12	272	12
3	Female	200	228	28	239	11
**Average (1)**		**217.3**	**237.3**	**20.0**	**248.0**	**10.7**
**Group 2**						
**1**	Female	229	254	25	261	7
**2**	Female	223	239	16	243	4
**3**	Female	251	270	19	273	3
**Average (2)**		**234.3**	**254.3**	**20.0**	**259.0**	**4.7**

(1) and (2) correspond to the administration of step 1 and step 2, respectively.

Table S6: Monthly arithmetic mean of minimum temperature, maximum temperature, temperature range, and monthly accumulated precipitation during the test period.

Month	Temperature			
	**Minimum (°C)**	**Maximum(°C)**	**Thermal Oscillation(°C)**	**Precipitation (mm)**
**October**	7.8	20.0	12.2	37.6
**November**	9.4	22.0	12.6	36.6
**December**	10.9	26.8	15.9	0.0
**January**	13.1	30.5	17.5	0.2

Table S7: Recording of temperatures and precipitation on the day of application.

Date	Temperature			Precipitation (mm)
	Minimum (°C)	Maximum (°C)	Thermal Oscillation (°C)	
**02-10-2023**	9.6	17.6	8.0	0.0
**11-10-2023**	7.8	20.1	12.3	0.0
**19-10-2023**	7.1	24.5	17.4	0.0
**31-10-2023**	7.4	19.3	11.9	0.0
**09-11-2023**	9.6	27.7	18.1	0.0
**23-11-2023**	8.4	27.5	19.1	0.0

Table S8: Evaluation of macroscopic pathologies (necropsy) in animals treated with 2000 mg/kg of active extract after 28 days.

Dose 2000 mg/kg	Females (1)			Females (2)		
	1	2	3	1	2	3
**Nervous**	-	-	-	-	-	-
**Respiratory**	L	-	-	-	-	-
**Digestive**	-	L	-	L	-	-
**Cardiovascular**	-	-	-	-	-	-
**Hematopoietic**	-	-	-	-	-	-
**Glandular**	-	-	-	-	-	-
**Urogenital**	-	-	-	-	-	-

(-): No abnormality observed; (P) Presence of abnormality: Degree of severity: (L) Mild. (M) Moderate and (S) Severe. (1) and (2) correspond to the administration of step 1 and step 2, respectively.

Table S9: Summary of clinical signs evaluated in rats.

**1. Nervous system and behavior**
Excessive grooming
Compulsive behavior: aggression
Seizures
Circular movements
Motor impairment (hyperreflexia or hyporeflexia)
Constant chewing
Paresis (muscle weakness)
**2. Respiratory system**
Breathing difficulties (dyspnea – hyperventilation)
Epistaxis (nosebleed)
Nasal secretions (mucus)
Pulmonary rales
Coughing/sneezing
**3. Digestive system**
Constipation
Diarrhea
Rectal prolapse
Excessive salivation
**4. Cardiovascular and hematopoietic system**
Anemia (paleness in ears and paws)
Cyanosis
Bleeding from natural orifices
**5. Skin and Sense Organs**
Blepharospasm
Pupillary reflex (mydriasis/miosis)
Profuse lacrimation
Bloody lacrimation (chromodacryorrhea)
Mucous membranes (pallor, cyanosis, or jaundice)
Crepitant skin
Piloerection
Corneal ulceration
**6. Urogenital system**
Anuria/polyuria
Vaginal prolapse
Preputial/vaginal seromucous discharge

Preputial/vulvar mucosal irritation
**7. Musculoskeletal system**
Kyphosis
Lordosis
Ataxia
Muscle spasticity
Lameness
Joint inflammation
Immobility
**8. Systemic compromise**
Cachexia
Coma
Dehydration
Edema
Sunken flanks
Prostration
Anorexia – Adipsia
Abdominal rigidity
Hyperthermia or hypothermia
